# Factor VII deficiency is more prevalent than previously reported

**DOI:** 10.1016/j.rpth.2025.103284

**Published:** 2025-12-05

**Authors:** Lukas Löwing Svensson, Elisabeth Aardal, Margareta Holmström

**Affiliations:** 1 Departments of Clinical Chemistry and Biomedical and Clinical Sciences, Linköping University, Linköping, Sweden; 2 Departments of Health, Medicine and Caring Sciences, and Acute Internal and Geriatric Medicine, Region Östergötland, Linköping University, Linköping, Sweden

**Keywords:** blood coagulation factors, factor VII deficiency, hemostatic disorders, prevalence, rare diseases

## Abstract

**Background:**

The prevalence of factor (F)VII deficiency is estimated at approximately 1 of 500,000 individuals. However, low FVII activity (FVII:C) is frequently observed in a population of <500,000 in the region of Östergötland, Sweden.

**Objectives:**

To estimate the prevalence and bleeding tendency of adult FVII-deficient patients in Östergötland, Sweden.

**Methods:**

In this retrospective cross-sectional study, the laboratory information system was used to identify adult patients with at least one FVII:C result ≤ 0.50 kilo International Units per Liter (kIU/L) between January 1, 2017, and December 31, 2022. The most likely cause was established, and in patients with a FVII deficiency, as defined by the Nordic Hemophilia Council (FVII:C < 0.35 kIU/L) or low FVII level (0.35-0.50 kIU/L), the bleeding tendency was evaluated.

**Results:**

FVII:C ≤ 0.50 kIU/L was identified in 97 patients residing in Östergötland. Of these, 21 had FVII deficiency, and 39 had low FVII level, corresponding to a prevalence of at least 1 of 17,800 and 1 of 9600 in the adult population in Östergötland, respectively. Patients with FVII deficiency differed significantly from patients with low FVII level in bleeding symptoms (*P* = .01). Of the FVII-deficient patients, 38% were asymptomatic, while 19% and 24% had experienced minor and major spontaneous bleedings, respectively.

**Conclusion:**

The prevalence of FVII deficiency was estimated to be at least 1 of 17,800 adults, which is 28 times higher than the internationally claimed prevalence. Spontaneous bleeding occurred in 43% of these individuals.

## Essentials

• FVII deficiency is a rare bleeding disorder with a reported prevalence of 1 in 500,000 individuals.
• This study used laboratory and patient records to identify cases and estimate bleeding tendency.
• FVII deficiency was found in 1 in 17,800 adults, which is higher than expected.
• Forty-three percent of FVII-deficient patients had experienced spontaneous bleeding episodes.

## 1 Introduction

Hemophilia A and B and von Willebrand disease are the most common inherited bleeding disorders [1]. Less frequent are deficiencies in other coagulation factors, such as fibrinogen, prothrombin, factor (F)V, FVII, FX, FXI, FXIII, the combined deficiencies of FV and FVIII, or vitamin K-dependent factors. The prevalence of these disorders is usually reported to range from 1 in 500,000 to 1 in 2,000,000 individuals [[1], [2], [3], [4]], with higher numbers in countries where consanguineous marriages are common due to the predominantly autosomal recessive inheritance pattern [4]. However, accurately estimating these figures is difficult given their rare nature [2]. FVII deficiency is among the most prevalent of the rare bleeding disorders, but is still reported to be only approximately 1 in 500,000 individuals [[1], [2], [3], [4]].

To improve the understanding of rare bleeding disorders, different registries have been established in recent years, including the North American Registry of Rare Bleeding Disorders [5], the European Network of Rare Bleeding Disorders [3], and the World Federation of Hemophilia Annual Global Survey [6]. In the latest edition of the latter, from 2023, hemophilia centers from 87 countries report a total of 17,453 patients with FVII deficiency [6]. However, these figures only include patients known to the respective hemophilia centers. Limited data are available from the Nordic countries. However, 39 patients were reported from Norway, corresponding to a prevalence of approximately 1 in 140,000 individuals.

Whereas symptoms of hemophilia A and B typically include muscular hematomas and joint bleeds, the most common bleeding symptoms in patients with rare factor deficiencies, including FVII deficiency, are epistaxis, menorrhagia, and bleeding during invasive procedures or childbirth [2]. In most bleeding disorders, there is an association between factor activity and bleeding tendency. However, this association is relatively weak for FVII and FV, and no such correlation has been found for FXI [3], which complicates the management of patients with these deficiencies.

The “*Practical Nordic guideline for diagnosis and management of FVII deficiency*,” [7] written by the Nordic working group on FVII deficiency under the Nordic Hemophilia Council, recommends that a FVII level < 0.35 kilo International Units per Liter (kIU/L) should be used to define FVII deficiency, while 0.35 to 0.50 kIU/L should be defined as a low FVII level. Although other studies have used similar cutoff values, there is no internationally accepted definition of which patients should be diagnosed with FVII deficiency. However, it is recognized that the laboratory method used to analyze FVII activity (FVII:C) is based on human tissue or recombinant thromboplastin as a clotting activator to ensure accurate measurements. Animal-derived thromboplastins have been shown to yield lower factor activities without a corresponding bleeding tendency [7].

Patients with FVII deficiency are usually diagnosed either as a result of investigation for a bleeding tendency, due to an unexpected finding of an elevated prothrombin time (PT) international normalized ratio (INR), or due to a family history in a proband. In the Swedish healthcare region of Östergötland, with 471,912 inhabitants as of December 31, 2022, approximately 1 patient with FVII deficiency is expected, given the claimed prevalence of 1 in 500,000 individuals with this disease. However, low FVII:C is often noted by laboratory physicians at the Department of Clinical Chemistry in Linköping, the only clinical laboratory in the healthcare region of Östergötland that analyzes FVII.

## 2 Objectives

The aim of this study was to estimate the prevalence of FVII deficiency in Östergötland by analyzing FVII results from the Department of Clinical Chemistry in Linköping, and to assess the extent to which patients with FVII deficiency experience bleeding symptoms by reviewing patient records.

## 3 Materials and Methods

The laboratory information system (LIS), Flexlab Lifecare (Tietoevry), in Östergötland was used to identify adult patients (age ≥18 years) with at least one FVII:C result ≤ 0.50 kIU/L and a sampling date between January 1, 2017, and December 31, 2022. During this period, FVII:C was measured using a one-stage clotting method with Dade Innovin reagent (Siemens Healthcare Diagnostics) and coagulation FVII-deficient plasma (Siemens Healthcare Diagnostics) on the Sysmex CS-2100 or CS-5100 instruments (Sysmex Corporation, distributed by Siemens Healthcare Diagnostics). The reagent is based on recombinant human tissue factor, as recommended by the Nordic Guidelines for FVII Deficiency [7].

For each identified patient, the referral information, test results, and referral response were reviewed to determine the most likely cause of the decreased FVII:C. When an obvious explanation other than isolated FVII deficiency was present, eg, ongoing warfarin treatment, vitamin K deficiency, or liver disease, further investigation was discontinued. For all other patients, an initial review was conducted to ensure they were residing in Östergötland at the time of blood sampling. Thereafter, the following data were extracted from either the LIS or the medical record, Cambio Cosmic (Cambio Healthcare Systems): age, sex (according to the Swedish personal identity number [odd penultimate digit for males, even for females]), lowest measured level of FVII:C, concurrent PT INR, FII, FIX, and FX activity during the period, reason for measuring FVII:C and most probable cause of decreased FVII:C, result from genetic investigation of the FVII gene, whether any International Classification of Diseases code relating to the low factor level was registered, patient history of bleeding, and contacts with coagulation consultants. Grading of bleeding symptoms was performed according to a 4-point scale used by Peyvandi et al. [3]: asymptomatic: no documented bleeding episodes; grade I: bleeding that occurred after trauma or drug ingestion (antiplatelet or anticoagulant therapy); grade II: spontaneous minor bleeding, including bruising, ecchymosis, minor wounds, oral cavity bleeding, epistaxis, and menorrhagia; and grade III: spontaneous major bleeding, including intramuscular hematomas requiring hospitalization, hemarthrosis, and central nervous system, gastrointestinal, and umbilical cord bleeding.

The cause of the decreased FVII:C was based on the assessment of either the physician in charge of the patient or the coagulation consultant involved in the case, unless another, more likely explanation emerged from other data. If ≥2 reasons were considered equally likely, the cause was interpreted as not a congenital FVII variant to obtain a conservative estimate of the prevalence. Patients with a likely congenital cause were assessed as having either FVII deficiency or a low FVII level according to their lowest FVII result, either during the sampling period or at the time before the study, as long as the same laboratory method had been used. The study was reviewed and approved by the Swedish Ethical Review Authority (2023-03499) and funded by the Department of Clinical Chemistry, Linköping, Sweden.

### 3.1 Statistical analysis

The number of adults residing in Östergötland, Sweden, was calculated by summing all inhabitants in Östergötland aged ≥18 years on December 31, 2022, from the Statistics Sweden Statistical Database [8]. The prevalence of FVII deficiency and low FVII levels was calculated by dividing the number of identified patients in each group by the number of adult inhabitants in Östergötland. The difference in bleeding tendency was calculated using Fisher’s exact test in R version 4.3.2 (R Core Team), with a significance level of *P* < .05.

## 4 Results

**Figure 1:**
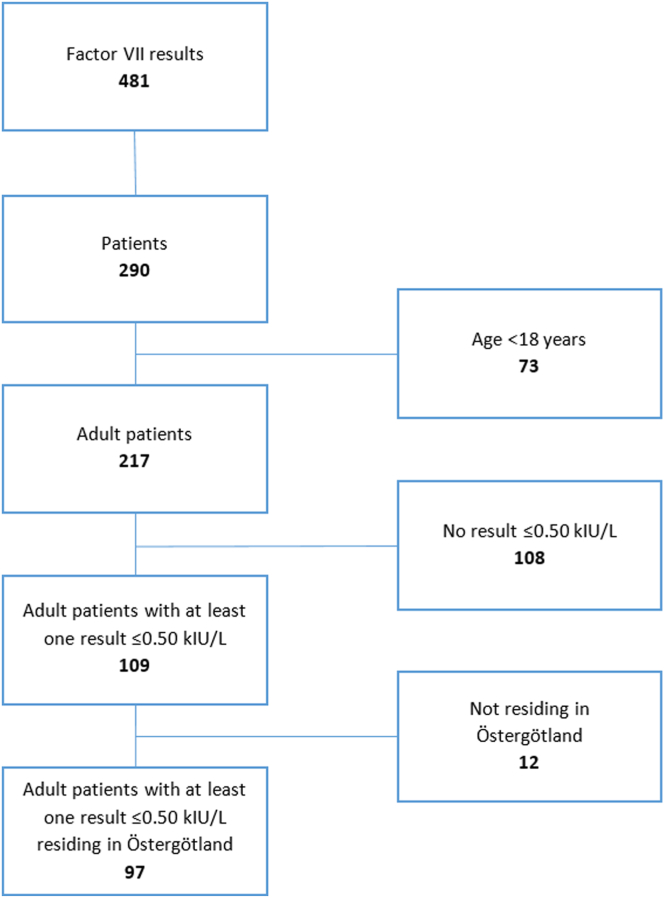
Decision tree for the inclusion of patients. kIU/L, kilo International Units per Liter.

The LIS contained 481 FVII results from 290 unique individuals during the period from January 1, 2017, to December 31, 2022. After excluding patients aged <18 years, 217 remained. Of these, 109 had at least one FVII:C result ≤ 0.50 kIU/L and were therefore included in the study. In an initial investigation, 12 patients were discovered as nonresidents of Östergötland at the time of blood sampling and were therefore excluded from further analysis (Figure 1).

Among the 97 remaining patients residing in Östergötland with at least one FVII:C result ≤ 0.50 kIU/L, the most common reason for referral for FVII testing was an elevated PT INR (*n* = 71), followed by a reported bleeding tendency (*n* = 19). Three patients were referred due to a family history of FVII deficiency, 1 patient was referred for assessment of warfarin treatment, and the referral information was not provided for 3 patients. The demographic data showed a small male majority (60%) but an even age distribution, with a median age of 55 years (Table 1).

**Table 1:** Sex and age characteristics of patients in Östergötland with factor VII activity ≤ 0.50 kIU/L.

Characteristic	Value
Sex, *n* (%)	
Male	58 (60)
Female	39 (40)
Age (y)	
Range	18-94
IQR	34-75
Median	55

IQR, interquartile range.

**Figure 2:**
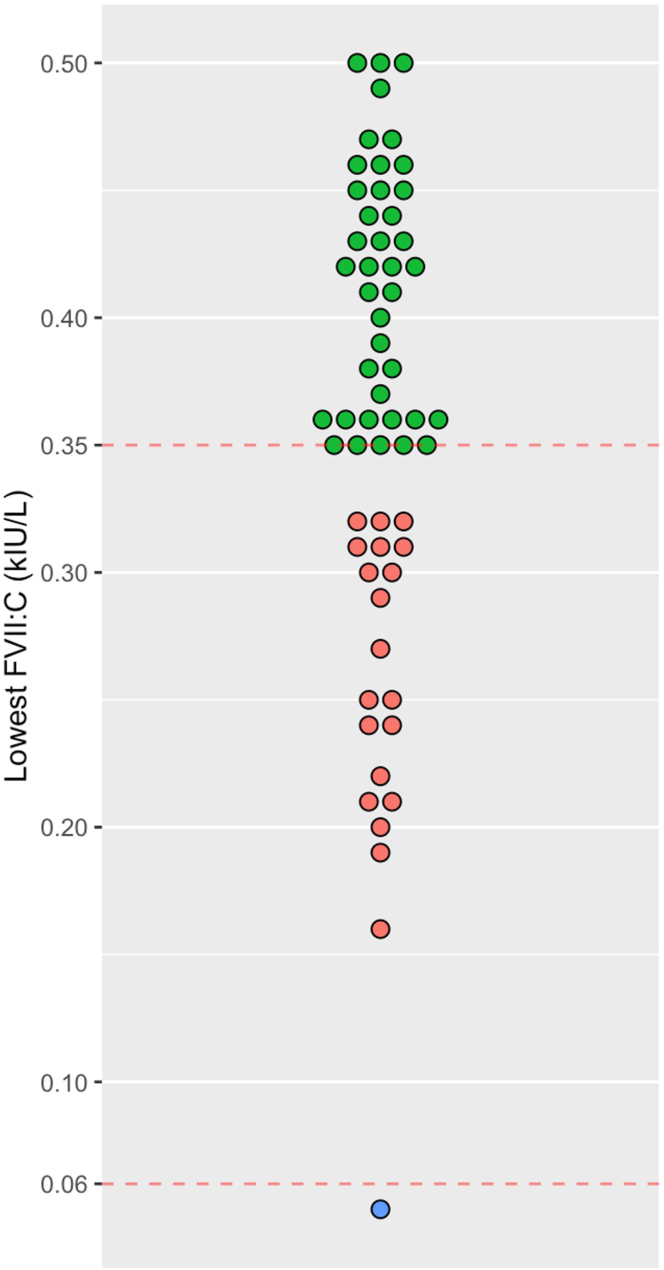
Lowest identified factor (F)VII activity (FVII:C) in the 60 patients with either low FVII:C (green) or FVII deficiency (red). One patient (indicated in blue) had a FVII:C < 0.06 kIU/L, below the method’s measuring range. kIU/L, kilo International Units per Liter.

For 16 of these 97 patients, referral information or the pattern of decreased coagulation factors (eg, all vitamin K-dependent factors decreased, stated liver cirrhosis/liver failure, or treatment with warfarin) made the finding of an isolated FVII deficiency highly implausible. They were therefore excluded from further investigation as to the cause of the decreased FVII:C. For the remaining 81 patients, for whom referral information or a pattern of decreased coagulation factors did not allow exclusion of an isolated FVII deficiency or low FVII:C, the medical records were reviewed. In 21 cases, other causes were considered responsible for the decrease, but in 60 cases, a likely congenital cause was identified (Table 2). Of these 60 cases, 21 patients met the diagnostic criteria according to the Nordic Guidelines for FVII Deficiency [7], with at least 1 value < 0.35 kIU/L, whereas 39 patients had their lowest value between 0.35 and 0.50 kIU/L (Figure 2). In one patient, FVII:C was slightly < 0.35 kIU/L (0.30 kIU/L) on only 1 of 4 occasions; she was therefore assigned to the low FVII group.

**Table 2:** Causes of decreased factor VII activity after review of medical records for 81 patients with possible factor VII deficiency.

Cause	All *N*	FVII:C < 0.35 kIU/L *n*	FVII:C 0.35-0.50 kIU/L *n*
Congenital cause	60	21	39
FVII inhibitors	1	1	0
Liver failure/cirrhosis	7	2	5
Vitamin K deficiency	5	1	4
Severe disease	6	2	4
Prescription drugs	2	0	2
Total	81	27	54

FVII, factor VII; FVII:C, factor VII activity; kIU/L, kilo International Units per Liter.

The adult population in Östergötland in 2022 was 374,632 according to Statistics Sweden [8]. The prevalence of FVII deficiency was thus 1 in 17,800 adults, and the prevalence of low FVII:C was 1 in 9600 adults. When grouped together, the prevalence of either diagnosis was 1 in 6200 adults during the study period.

The bleeding tendency in patients with FVII deficiency or low FVII levels was identified through a review of the medical records and summarized in Table 3. Patients with FVII deficiency differed significantly from those with low FVII levels (*P* = .01). After reclassification of 2 patients with possible other causes of bleeding into the asymptomatic group (see Table 3 legend), the result was still significant (*P* = .04). Only a minority of patients with FVII deficiency were asymptomatic (38%), while 19% and 24% had experienced spontaneous minor and major bleeding episodes, respectively.

**Table 3:** Bleeding tendency among patients with factor VII deficiency or decreased factor VII activity.

Bleeding tendency	All *N* (%)	FVII:C < 0.35 kIU/L *n* (%)	FVII:C 0.35-0.50 kIU/L *n* (%)
Asymptomatic	32 (53)	8 (38)	24 (62)
Grade I	6 (10)	4 (19)	2 (5)
Grade II	16 (27)	4 (19)	12 (31)^a^
Grade III	6 (10)	5 (24)^b^	1 (3)
Total	60 (100)	21 (100)	39 (100)

Asymptomatic, no documented bleeding episodes; FVII:C, factor VII activity; Grade I, bleeding that occurred after trauma or drug ingestion (antiplatelet or anticoagulant therapy); Grade II, spontaneous minor bleeding, including bruising, ecchymosis, minor wounds, oral cavity bleeding, epistaxis, and menorrhagia; Grade III, spontaneous major bleeding, including intramuscular hematomas requiring hospitalization, hemarthrosis, and central nervous system, gastrointestinal, and umbilical cord bleeding.

kIU/L, kilo International Units per Liter.

a One patient with simultaneous platelet dysfunction.

b One patient with simultaneous severe thrombocytopenia, platelets 2 × 10^9^/L.

In 51 of 60 cases, a coagulation consultant was involved in the diagnosis or follow-up of patients with FVII deficiency or low FVII:C. Of these, 39 had at least one visit to a coagulation clinic, and 6 were followed regularly.

## 5 Discussion

In this study, 21 adult patients residing in Östergötland, Sweden, were identified as fulfilling the criteria for FVII deficiency according to the Nordic Guidelines for FVII Deficiency [7], whereas 39 patients were classified as having low FVII levels. This corresponds to a prevalence of FVII deficiency in the adult population of Östergötland of 1 in 17,800 individuals, which is 28 times higher than the internationally reported prevalence of 1 in 500,000. The bleeding tendency among patients with FVII deficiency differed significantly from that observed in patients with low FVII levels. Only 38% of those with FVII deficiency were asymptomatic, while 19% and 24% had experienced minor and major spontaneous bleeding episodes, respectively.

### 5.1 Prevalence estimation

The comparison of prevalence of FVII deficiency across different studies is difficult, given that there is no universally accepted international definition of the condition. One strength of this study is that we opted to use a well-established definition across the Nordic countries and a moderately conservative diagnostic procedure. Patients with ≥2 equally likely diagnoses were not classified as having a congenital cause of FVII deficiency. However, in 7 of 21 patients with FVII deficiency and 5 of 39 patients with low FVII levels, only a single FVII measurement was recorded, despite the recommendation from the laboratory to confirm low FVII:C with a follow-up sample. Nevertheless, we believe the diagnosis of FVII deficiency can be established with a high level of certainty when clinical presentation, results of simultaneous measurements of FII and FX (and, in some cases, FIX), and other data in the patient’s records are taken into account. In almost all patients without repeated measurements, the elevated PT INR persisted over time.

The observed prevalence of FVII deficiency is almost certainly an underestimate of the true number. Since follow-up testing is rarely conducted in individuals with no or mild bleeding symptoms, many previously diagnosed patients may not have had a recorded FVII measurement during the study period. As a result, this cohort consists largely of newly diagnosed cases, while previous cases and asymptomatic individuals who have not yet been diagnosed are not accounted for. This hypothesis is supported by the fact that only a small proportion of the included patients had a FVII measurement before the study period and were therefore diagnosed for the first time between 2017 and 2022. The prevalence of FVII deficiency and low FVII levels in Östergötland would thus probably be higher if a longer study period had been used. Although the short study duration is a limitation, with the risk of potential underestimation of the prevalence, it also represents a strength, as it permitted a thorough review of the records of all included patients and ensured continuity in the laboratory method used.

The observed prevalence may be due to a regionally prevalent FVII variant; however, it is more likely explained by differences in testing routines. Most patients were identified through a slightly elevated PT INR (as a screening test before initiation of certain medications, a planned surgical procedure, or, in some cases, because of a bleeding tendency). When the elevated PT INR was included in a laboratory bleeding investigation, a laboratory physician decided to measure FII, FVII, and FX if the activated partial thromboplastin time was within the normal range. In other cases, the attending physician directly requested FVII measurement in the referral. These routines resulted in 217 adult patients being tested for FVII deficiency over the 7-year period. Children were not included due to a lack of established reference ranges for FVII:C. A higher frequency of PT INR measurements and a tendency to search for FVII deficiency in slightly elevated samples compared with other parts of the world might explain the observed high prevalence in Östergötland, Sweden. This is supported by prevalence figures from other European countries in the World Federation of Hemophilia’s Report on the Annual Global Survey from 2023 [6], which describe reported cases of FVII deficiency from, for example, Slovakia (*N* = 1029), Hungary (*N* = 471), and Portugal (*N* = 484), at similar prevalence estimates as in the present study. Despite these registry data, the internationally accepted prevalence is still reported as 1 in 500,000 individuals, even in the latest literature.

Since genetic testing is recommended only in clinically severe cases or for prenatal counseling according to the Nordic Guidelines for FVII Deficiency [7], only one patient identified in this retrospective study has, to our knowledge, been genotyped. To better understand the cause of the high prevalence and to confirm the congenital cause, further studies involving genotyping might be of interest to our cohort.

### 5.2 Bleeding symptoms

The bleeding tendency of the 21 FVII-deficient patients in our study differed significantly from that of the 39 patients with slightly higher levels (FVII:C 0.35-0.50 kIU/L). Several authors have noted a weaker correlation between FVII:C and bleeding diathesis than with many other coagulation factors [3]. Nevertheless, studies have demonstrated correlations between bleeding symptoms and factor activity and zygosity (heterozygous, double heterozygous, or homozygous), as well as between factor activity and bleeding tendency in patients undergoing surgery [[9], [10], [11], [12]].

Herrmann et al. [11] studied 717 genotyped patients with a causative F7 variant and FVII:C of < 0.01 to 0.50 kIU/L in index patients. Seventy-one percent of homozygotes (mean FVII:C, 0.05 kIU/L), 50% of double heterozygotes (mean FVII:C, 0.06 kIU/L), and 6% of heterozygotes (mean FVII:C, 0.46 kIU/L) were found to be symptomatic. Of these patients, 539 were inhabitants of Germany, with a current population of approximately 80 million, indicating a higher prevalence of 1 in 500,000 individuals when using a more inclusive definition of factor activity than in our study. Severe bleeding episodes (gastrointestinal or intracranial hemorrhages) were seen only in homozygotes and double heterozygotes.

A working group from the International Society on Thrombosis and Haemostasis published a study in 2012 that used data from several registries, as well as published cases with clinical and laboratory data [12]. Clinical severity was classified into 3 levels, with factor activity associated with each specific deficiency. For FVII, FVII:C < 0.10 kIU/L was proposed to correlate with severe disease (associated with spontaneous major bleeding), FVII:C of 0.10 to 0.20 kIU/L was proposed to correlate with moderate disease (associated with spontaneous or triggered bleeding), while FVII:C > 0.20 kIU/L was suggested to correlate with mild disease (mostly asymptomatic). The largest registry included in this study was the European Network of Rare Bleeding Disorders. When Peyvandi et al. [3] analyzed 224 patients with FVII deficiency from this registry, using the bleeding classification later used in our study, they found that 55% were asymptomatic, whereas 17%, 22%, and 7% had grade I, II, and III bleeding symptoms, respectively. The mean FVII:C associated with each grade was 0.25 kIU/L (95% CI, 0.15-0.35), 0.19 kIU/L (95% CI, 0.08-0.30), 0.13 kIU/L (95% CI, 0.02-0.25), and 0.08 kIU/L (95% CI, 0.00-0.21), respectively [3]. The proportion of patients in each bleeding category was similar to our findings, except that Peyvandi et al. [3] found a lower proportion of patients with grade III symptoms (7% vs 24%). This difference is most likely due to the strict inclusion criterion of only patients with FVII levels < 0.35 kIU/L in our study and the uncertainties associated with the small number of patients. In addition, one patient with grade III bleeding in our study also had concomitant severe thrombocytopenia, which most likely contributed to major bleeding. Excluding this patient from the most severe bleeding grade would reduce the proportion of patients with grade III symptoms to 19%.

When scrutinizing all patients with FVII deficiency or low FVII level (ie, FVII:C 0.00-0.50 kIU/L) in our study, 53% were asymptomatic, while 10%, 27%, and 10% presented with grade I, II, and III bleeds, respectively. These figures are consistent with the results of the study by Peyvandi et al. [3], which indicates that the patients included in our study are similar to those previously diagnosed in Europe.

### 5.3 Further studies

Genotyping the patients in this study and comparing FVII variants with those reported in other registries may shed some light on explaining the high prevalence findings and help differentiate between testing routines and genetic factors as the cause. By extending the study period and searching for patients with corresponding International Classification of Diseases codes in the patient records, additional patients with factor deficiencies could be identified, and a better approximation of the true prevalence could be obtained.

## 6 Conclusion

In Östergötland, Sweden, the prevalence of FVII deficiency was estimated to be at least 1 in 17,800 adults, which is 28 times higher than the internationally reported prevalence. Forty-three percent of the identified patients had experienced minor or major spontaneous bleedings, and only 38% were asymptomatic.
